# Are Dieting and Dietary Inadequacy a Second Hit in the Association with Polycystic Ovary Syndrome Severity?

**DOI:** 10.1371/journal.pone.0142772

**Published:** 2015-11-16

**Authors:** Nicole A. Huijgen, Joop S. E. Laven, Chantal T. Labee, Yvonne V. Louwers, Sten P. Willemsen, Régine P. M. Steegers-Theunissen

**Affiliations:** 1 Department of Obstetrics and Gynaecology, Erasmus MC, University Medical Centre, Rotterdam, The Netherlands; 2 Department of Obstetrics and Gynaecology, Division of Reproductive Medicine, Erasmus MC, University Medical Centre, Rotterdam, The Netherlands; 3 Department of Biostatistics, Erasmus MC, University Medical Centre, Rotterdam, The Netherlands; Suzhou University, CHINA

## Abstract

**Background:**

The composition of the diet is of increasing importance for the development and maturation of the ovarian follicles. In Polycystic Ovary Syndrome (PCOS) healthy dietary interventions improve the clinical spectrum. We hypothesized that dieting and diet inadequacy in the reproductive life course is associated with impaired programming of ovarian follicles and contributes to the severity of the PCOS phenotype.

**Methods and Findings:**

To determine associations between the use of a self-initiated diet and diet inadequacy and the severity of the PCOS phenotype, we performed an explorative nested case control study embedded in a periconception cohort of 1,251 patients visiting the preconception outpatient clinic. 218 patients with PCOS and 799 subfertile controls were selected from the cohort and self-administered questionnaires, anthropometric measurements and blood samples were obtained. The Preconception Dietary Risk Score (PDR score), based on the Dutch dietary guidelines, was used to determine diet inadequacy in all women. The PDR score was negatively associated to cobalamin, serum and red blood cell folate and positively to tHcy. PCOS patients (19.9%), in particular the hyperandrogenic (HA) phenotype (22.5%) reported more often the use of a self-initiated diet than controls (13.1%; p = 0.023). The use of an inadequate diet was also significantly higher in PCOS than in controls (PDR score 3.7 vs 3.5; p = 0.017) and every point increase was associated with a more than 1.3 fold higher risk of the HA phenotype (adjusted OR 1.351, 95% CI 1.09–1.68). Diet inadequacy was independently associated with the anti-Müllerian Hormone (AMH) concentration (β 0.084; p = 0.044; 95% CI 0.002 to 0.165) and free androgen index (β 0.128; p = 0.013; 95% CI 0.028 to 0.229) in PCOS patients.

**Conclusions:**

The use of a self-initiated diet and diet inadequacy is associated with PCOS, in particular with the severe HA phenotype. This novel finding substantiated by the association between diet inadequacy and AMH needs further investigation.

## Introduction

Polycystic Ovary Syndrome (PCOS) is the most common endocrine disorder affecting young women of reproductive age, resulting in subfertility and increased risk of cardiovascular related disease in later life [1]. The estimated prevalence of PCOS varies between 5–15% [2,3]. PCOS is characterized by oligo- or anovulation (ovulatory dysfunction; OD), clinical and/or biochemical signs of hyperandrogenism (HA) and/or polycystic ovaries (Polycystic ovarian morphology; PCOM), and gene-environment interactions play a significant role in the variability within PCOS phenotypes [4,5]. The clinical spectrum and severity in PCOS is heterogeneous, including reproductive, metabolic and psychological features. Because of the profound cardiovascular and metabolic disturbances in hyperandrogenic PCOS patients, it is recommended to distinguish two subtypes of PCOS according to the severity of disease (HA-PCOS and non HA-PCOS) [6].

Personal behaviors, such as dieting, smoking, alcohol- and drug use, stress and exercise are considered behavioral factors that are important for reproductive functioning [7,8]. The composition of the preconception diet is of increasing interest, because over- and undernutrition derange metabolic and endocrine pathways, especially the B-vitamin dependent one-carbon (1-C) pathway [8–10]. During the development of the germ cells and the maturation of the ovarian follicles, the methyl groups mainly derived from B vitamins, methionine and choline are used for DNA synthesis and phospholipid and protein biosynthesis. Deficiencies of these nutrients are inversely associated with ovarian follicle development, the number of oocytes retrieved for IVF treatment, embryo quality and pregnancy outcome [8–10]. Worrisome is that in more than 50% of subfertile couples planning pregnancy the preconception diet is inadequate [11]. However it is promising that improvement of the diet can increase the chance of ongoing pregnancy up to 65% [12]. The prevalence of obesity and PCOS is increasing and although obesity is not the cause of PCOS it contributes to the clinical spectrum [13]. Moreover, weight loss can improve the clinical spectrum including menstrual regularity, insulin resistance and quality of life [14] and dietary interventions are therefore part of the clinical treatment of PCOS.

In the polycystic ovary, an arrest of follicular maturation results in the accumulation of small antral follicles that produce elevated levels of Anti-Müllerian Hormone (AMH) [15]. AMH concentration is therefore a marker of PCOS severity which is inversely associated with a healthy diet, irrespective of weight loss [16].

Psychological co-morbidities such as depression, eating and anxiety disorders, which can affect the nutritional state are also more prevalent in PCOS [17]. Another potential driver for differences in dieting could be obesity although previous research showed that dieting behavior is independent of current BMI, but strongly associated with negative emotions and problematic behaviors [18]. Dietary behavior is acquired during childhood and remains rather stabile over time only varying during episodes of illnesses, dieting and increased needs [19–22]. Dieting habits can be transferred unknowingly since the practices mothers adopt predict the children’s diet quality [23] and because parents contribute to adolescents' motivation to diet [24]. Here we hypothesize that regular self-initiated dieting and using an inadequate diet is associated with alterations in the 1-C pathway and with impaired functioning of ovarian follicles which contributes to the clinical spectrum of PCOS. We performed a case-cohort study embedded in a prospective periconception cohort study of couples referred for subfertility, to investigate whether the use of a self-initiated diet and dietary inadequacy are associated with PCOS severity and phenotype.

## Materials and Methods

### Study design

Between October 2007 and March 2011, all couples planning pregnancy and visiting the outpatient clinic of the Department of Obstetrics and Gynaecology at the Erasmus MC, University Medical Centre Rotterdam, a tertiary hospital in the Netherlands, were offered nutrition and lifestyle counselling at the preconception outpatient clinic ‘Achieving a Healthy Pregnancy’ [11,12].

In this study women completed a questionnaire from which we extracted general characteristics, such as age, ethnicity (Dutch or Non-Dutch), educational level (low, intermediate or high), the use of a diet (energy restricted, vegetarian, macrobiotic, vegan, other) and of six main food groups (Preconception Dietary Risk score), folic acid supplements (yes/no), vitamin supplements (yes/no), medication (yes/no), alcohol (yes/no), and smoking (yes/no), physical exercise (yes/no), and the experience of stress (yes/no). Ethnicity and education level were classified according to the definition of Statistics Netherlands (www.cbs.nl; 2012). The validated summary PDR score, based on the Dutch food based dietary guidelines was used to assess diet inadequacy in the outpatient clinical setting [12,25]. When patients did not meet the dietary guidelines for the food group, one point was administered. Since six guidelines were evaluated, the PDR score had a maximum of 6 points and a minimum of 0 points. Thus, the higher the score the more inadequate the intake according to the guideline. The adequate intake per food group was defined without a specific time frame by the following guidelines: 4–5 slices of whole wheat bread daily, the use of monounsaturated or polyunsaturated oils/fats, 200 grams of vegetables daily, 2 pieces of fruit daily, 3–4 servings of meat weekly and 2 servings of fish weekly [26]. During the visit at the outpatient clinic the questionnaires were verified by a trained counsellor.

Subsequently non-fasting blood samples were obtained by venipuncture and anthropometrics (i.e. height, weight and circumferences) were measured. Patients with oligo- or anovulation were screened for ovulatory dysfunction by trained professionals according to a standardized protocol.

The diagnosis of PCOS was based on the Rotterdam criteria [5] including the presence of oligo- or anovulation (i.e. menstrual bleeding interval between 35–182 days or absence of menstrual bleeding for more than 182 days), hyperandrogenism (Ferriman-Gallwey score ≥ 9 and/or a Free Androgen Index > 4.5), and/ or polycystic ovaries (volume exceeding 10 cm^3^ and/or the follicle count was ≥ 12).

### Study population

**Fig 1:**
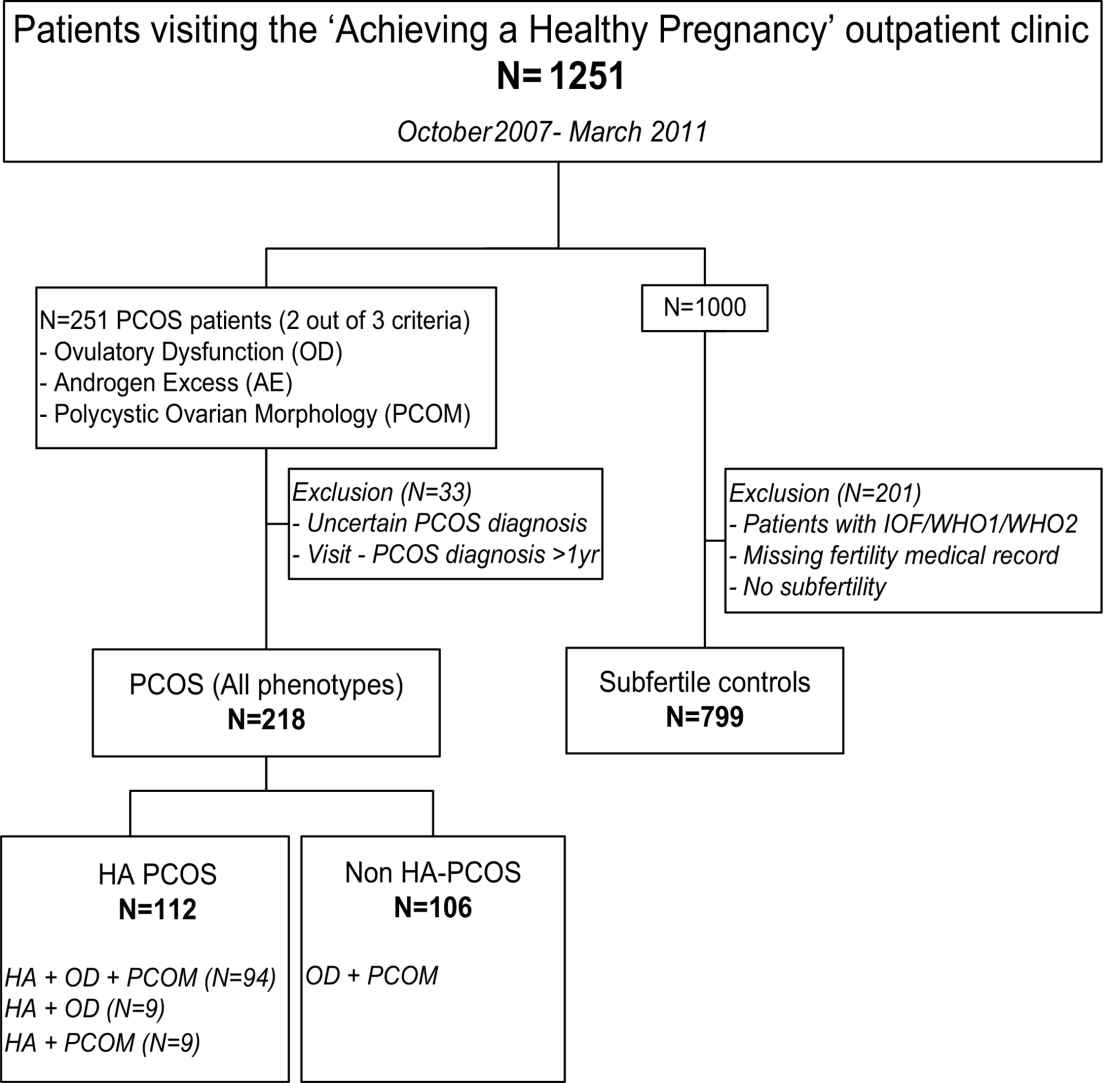
Flowchart of the selection of the study population. IOF = imminent ovarian failure; WHO1 = hypogonadotropic hypogonadal anovulation; WHO2 = normogonadotropic normoestrogenic anovulation; HA = hyperandrogenic.

During the study period 1,348 couples visited the preconception outpatient clinic of which 1,251 participated in the study and provided a written informed consent. Two hundred and fifty one patients were diagnosed with PCOS and 33 patients were excluded because of an uncertain diagnosis or the period between enrolment and PCOS diagnosis comprised more than one year. From the remaining 1,000 patients we excluded patients with an incomplete fertility medical record (n = 80), patients with ovulatory disorders such as imminent ovarian failure, WHO 1 or WHO 2 disorders (n = 107) and fertile patients (n = 14) who were referred because of a complicated obstetrical history. This resulted in a control group of 799 subfertile patients (Fig 1). According to phenotype, we divided the PCOS group (n = 218) in the HA (n = 112) and non HA (n = 106) phenotype.

### Biomarkers

On the day of the visit at the outpatient clinic blood samples were drawn and processed within 2 hours after withdrawal. Serum was stored at −20°C until assayed. In blood the biomarkers of 1-C metabolism serum and red blood cell (RBC) folate, serum cobalamin, and plasma total homocysteine (tHcy) were determined in all study participants. tHcy was measured using HPLC-TandemMS. For the determination of serum folate and cobalamin an immunoelectrochemoluminescence assay was used (E170; Roche Diagnostics GmbH, Mannheim, Germany) and for the determination of RBC folate the ADVIA 120 Hematology Analyzer was used (Bayer Diagnostics, Leverkusen, Germany).

For clinical purposes the patients with PCOS were additionally screened on hormonal and metabolic parameters. Testosterone was measured using radioimmunoassay (RIA) (Siemens DPC, Los Angeles, CA) and glucose levels were measured by using a Hitachi 917 analyzer (Roche Diagnostics, Almere, The Netherlands). The Free Androgen Index (FAI) was calculated as follows; 100 x [T (nmol/L) / Sex hormone-binding globulin (SHBG) (nmol/L)]. An immunoluminometric assay (Immulite^®^ platform, Siemens Diagnostic Product Corporation, Los Angeles, CA) was used to measure LH, FSH, SHBG, androstenedione, insulin and DHEAS. Serum AMH (Anti-Müllerian Hormone) levels were measured by using an in-house double-antibody ELISA (GenII; Beckman Coulter) as previously described [27]. Intra- and interassay coefficients of variation were, respectively, <5% and <15% for LH, <3% and <18% for FSH, <3% and >5% for T, <8% and >11% for AD, <4% and >5% for SHBG, <9% and >11% for DHEAS, <5% and >8% for AMH, and <6% and >8% for insulin.

### Statistical analyses

Continuous variables are presented as median with interquartile range. Dichotomous and categorical variables are presented as count and proportions. Chi Square tests with comparison of column proportions were performed with a Bonferroni correction to compare the distribution of categorical variables between PCOS phenotypes and subfertile controls. Mann Whitney U Tests were performed to compare the total group of PCOS with the subfertile controls. Kruskal Wallis tests with pairwise comparisons adjusted for the number of pairwise tests were performed for comparison of controls, HA-PCOS patients and non HA-PCOS patients. Simple linear regression analysis were conducted to establish associations between the PDR score and the biomarkers of 1-C metabolism. To achieve normality a natural log transformation was performed for cobalamin, RBC Folate and tHcy and a square root transformation for serum folate. Multivariable logistic regression analysis was applied to calculate the risk of PCOS after one point increase of the PDR score for each phenotype separately with adjustment for the potential confounders body mass index (BMI), age, educational level, ethnicity, medication use and vitamin supplement use. These covariates were selected because of the significant association with the PDR score or when significant differences revealed between PCOS and controls. Multivariable linear regression analysis were performed to investigate associations between the PDR score and hormonal parameters in PCOS only with adjustments. Statistical analyses were performed using SPSS software for Windows (version 21.0, IBM SPSS, Statistics for Windows, Armonk, NY: IBM Corp). The level of significance was set to 0.05 for all analyses.

### Ethical approval

All questionnaire data and materials were processed anonymously. This study was conducted according to the guidelines of the Declaration of Helsinki, and all procedures involving patients were approved by the Medical Ethical and Institutional Review Board at the Erasmus MC, University Medical Centre in Rotterdam, the Netherlands. Written informed consent was obtained from all patients.

## Results

To evaluate selection bias we present the general characteristics of the included and excluded patients in S1 Table. The general characteristics were comparable in both groups except the slightly younger age, higher percentage of smoking and alcohol use and slightly smaller waist circumference and waist-hip ratio in the study group.

### PCOS study population

In Table 1 the general characteristics of PCOS patients (n = 218) and subfertile control patients (n = 799) are shown. The PCOS group was significantly younger and used less often alcohol, folic acid and vitamin supplements than subfertile controls, which is validated with the lower concentrations of serum and RBC folate observed in the PCOS group. They also used more often a self-initiated diet and were more frequently obese (BMI≥30).

**Table 1:** Preconception general characteristics.

	Total PCOS (n = 218) (Group A)	HA-PCOS (n = 112) (Group A1)	non HA-PCOS (n = 106) (Group A2)	Subfertile controls (n = 799) (Group B)	P value (Group A, B)	P value (Group A1, A2, B)
**Age** (years)	28.5 (25.5–31.3)	27.7 (24.5–30.0)^a^	29.0 (27.2–32.2)^a^	33.1 (29.6–36.4)^b^	**<0.01**	**<0.01**
**Ethnicity**						
Dutch	122 (56.0%)	54 (48.2%)	68 (64.2%)	445 (56.0%)	1.00	0.06
Other	96 (44.0%)	58 (51.8%)	38 (35.8%)	350 (44.0%)	-	-
**Educational level**						
Low	30 (14.6%)	20 (18.7%)	10 (10.1%)	120 (15.8%)	0.75	0.09
Intermediate	97 (47.1%)	55 (51.4%)	42 (42.4%)	336 (44.2%)	-	-
High	79 (38.3%)	32 (29.9%)	47 (47.5%)	304 (40.0%)	-	-
**Lifestyle Parameters:**						
Diet (Yes)	43 (19.9%)	25 (22.5%)^b^	18 (17.1%)^a, b^	105 (13.1%)^a^	**0.01**	**0.02**
PDR score (mean; sd)	3.7 (1.14)	3.9 (1.12)^a^	3.5 (1.13)^b^	3.5 (1.13)^b^	**0.02**	**<0.01**
Folic acid supplement use (No)	92 (42.4%)	55 (49.1%)^b^	37 (35.2%)^a, b^	268 (33.5%)^a^	**0.02**	**<0.01**
Vitamin supplement use (No)	145 (67.4%)	78 (70.3%)	67 (64.4%)	471 (59.5%)	**0.03**	0.07
Medication use (Yes)	75 (34.6%)	41 (36.6%)	34 (32.4%)	255 (32.0%)	0.48	0.63
Alcohol (Yes)	101 (46.3%)	39 (34.8%)^b^	62 (58.5%)^a^	439 (54.9%)^a^	**0.02**	**<0.01**
Smoking (Yes)	51 (23.5%)	29 (26.1%)	22 (20.8%)	175 (22.3%)	0.70	0.59
Physical exercise (No)	98 (51.0%)	56 (60.2%)^a^	42 (42.4%)^b^	366 (49.4%)^a, b^	0.68	**0.04**
Stress (Yes)	64 (34.4%)	30 (33.7%)	34 (35.1%)	245 (33.6%)	0.83	0.96
**Measurements:**						
BMI (kg/m2)	25.6 (22.0–31.2)	29.2 (24.9–33.2)^a^	23.1 (20.6–26.6)^b^	24.5 (22.0–28.3)^c^	**0.03**	**<0.01**
BMI categories:						
<20	21 (9.6%)	3 (2.7%)^a^	18 (17.0%)^b^	54 (6.8%)^a^	**<0.01**	**<0.01**
≥20 <25	82 (37.6%)	27 (24.1%)^b^	55 (51.9%)^a^	373 (47.0%)^a^	-	-
≥25 <30	52 (23.9%)	31 (27.7%)^a^	21 (19.8%)^a^	228 (28.8%)^a^	-	-
≥30	63 (28.9%)	51 (45.5%)^b^	12 (11.3%)^a^	138 (17.4%)^a^	-	-
Waist circumference (cm)	85 (74–97)	93 (83–105)^a^	77 (71–89)^b^	84 (75–93)^c^	0.28	**<0.01**
Waist hip ratio	0.81 (0.75–0.87)	0.84 (0.80–0.91)^a^	0.78 (0.74–0.83)^b^	0.81 (0.75–0.87)^c^	0.66	**<0.01**
**Biochemical Parameters:**						
Cobalamin (pmol/L)	305 (232–392)	282 (215–371)	322 (254–416)	310 (237–411)	0.51	0.09
RBC Folate (nmol/L)	960 (769–1182)	861 (718–1067)^a^	1059 (869–1380)^b^	1037 (831–1328)^b^	**<0.01**	**<0.01**
Folate (nmol/L)	25.1 (15.6–36.1)	21.3 (13.4–30.9)^a^	29.1 (17.8–43.4)^b^	30.1 (19.0–41.3)^b^	**<0.01**	**<0.01**
tHcy (μmol/L)	8.8 (7.4–10.2)	8.5 (7.3–10.2)	9.1 (7.7–10.2)	8.4 (7.1–9.9)	0.08	0.12

Note: Values are expressed as median (interquartile range) or number (%). HA = Hyperandrogenic; PDR score = Preconception Dietary Risk score; BMI = Body Mass Index; RBC Folate = Red Blood Cell Folate; tHcy = homocysteine. Normal range biochemical parameters; Folate ≥8 nmol/L, RBC folate ≥500 nmol/L, Cobalamin ≥145 pmol/L, tHcy <15 μmol/L. For pairwise comparisons; each subscript letter denotes a subset of categories whose column proportions do not differ significantly from each other.

### PCOS phenotypes

In Table 1 we depict the PCOS group divided into the HA (n = 112) and non HA phenotype (n = 106) and compare the general characteristics with subfertile controls (n = 799). The HA and non HA phenotype patients were both significantly younger than subfertile controls. Patients with the HA phenotype exercised less than non HA-PCOS patients and they used less often alcohol than non HA-PCOS patients or subfertile controls. Patients with the HA phenotype were also more likely to use a self-initiated diet and used less often folic acid supplements than subfertile controls and showed a significantly higher BMI and waist circumference compared to the non HA phenotype and subfertile controls. Moreover, the waist hip ratio, used as indicator of central obesity, was significantly lower in the non HA phenotype, compared to the HA phenotype and subfertile controls. Patients with the HA phenotype showed lower levels of serum and RBC folate than the non HA phenotype and subfertile controls.

### Dietary inadequacy assessed with the PDR score

**Fig 2:**
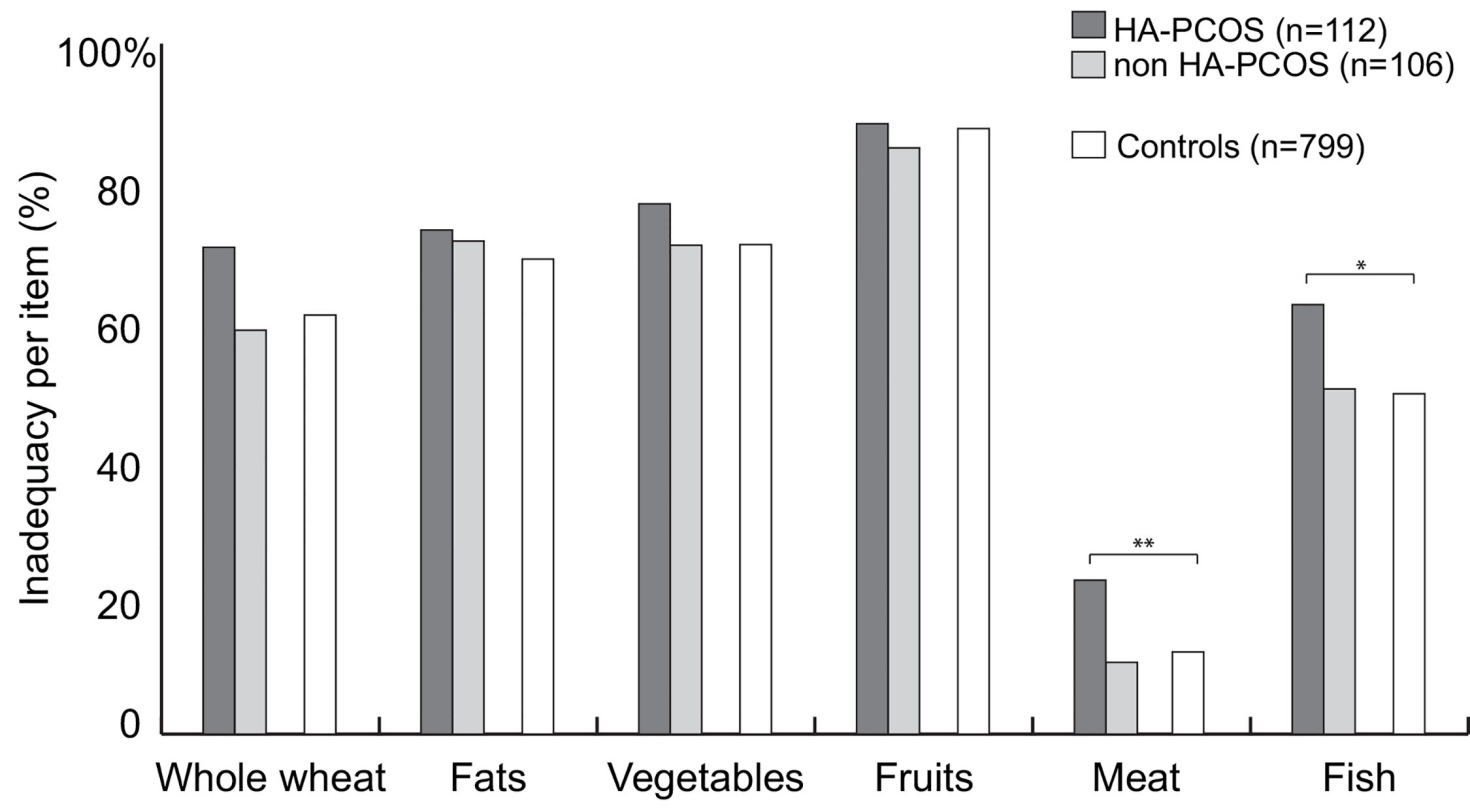
The items of the Preconception Dietary risk score. Depicted are the percentages of inadequate dietary intake for each food group. Meat* and fish** intake differed significantly between the controls, HA and non HA phenotype (respectively p 0.006 and p 0.039).

A higher mean PDR score was observed in the PCOS group than in the subfertile controls (3.7 vs 3.5; p = 0.017). Patients with the HA phenotype also showed a higher mean PDR score (3.9; p <0.001) than the non HA phenotypes (3.5) and the subfertile controls (3.5). The PDR score per food group was comparable between the groups, except for a higher percentage of inadequate meat and fish intake in the HA phenotype (Fig 2).

Table 2 shows that one point increase in the PDR score was associated with a 1.4 fold higher risk of the HA phenotype (unadjusted OR 1.427, 95% CI 1.183 to 1.721). The PDR score was inversely associated with age (β -0.037, 95% CI -0.051 to -0.023, p = <0.001), educational level (category high β -0.311, 95% CI -0.522 to -0.100, p = 0.004) and the use of vitamin supplements (β -0.192, 95% CI -0.336 to -0.048, p = 0.009) and therefore these covariates were selected for adjustment of the analyses. Age was a confounder and inversely associated with the severity of the PCOS phenotype and positively associated with the PDR score. After adjustment in model 1 the risk of the HA phenotype slightly increased (OR 1.520, 95% CI 1.240 to 1.863) and attenuated in model 2 (OR 1.351, 95% CI 1.087 to 1.679), but remained significant. The PDR score was not associated with the risk of the non HA phenotype.

**Table 2:** Multivariable logistic regression analyses; The risk of PCOS after one point increase in the Preconception Dietary Risk score compared to subfertile controls.

	HA-PCOS			non HA-PCOS		
	OR	P value	95% CI	OR	P value	95% CI
**Unadjusted:**	1.427	0.000	1.183–1.721	0.978	0.805	0.817–1.170
**Model I:** Adjusted for BMI, ethnicity, education, vitamin supplement use and medication use	1.520	0.000	1.240–1.863	0.937	0.520	0.767–1.143
**Model II:** Adjusted for age, BMI, ethnicity, education, vitamin supplement use and medication use	1.351	0.007	1.087–1.679	0.866	0.176	0.703–1.067

HA = Hyperandrogenic; OR = Odds Ratio; CI = Confidence Interval; BMI = Body Mass Index.

### The PDR score and biomarkers of 1-C metabolism

To investigate the association between dietary inadequacy and the markers of 1-C metabolism, we subsequently performed linear regression analyses in the PCOS patients and subfertile controls with the separate biomarkers cobalamin, RBC folate, serum folate and tHcy. A negative linear association was established between the PDR score and cobalamin (unadjusted β -0.052; p < 0.001; 95% CI -0.074 to -0.030), RBC folate (unadjusted β -0.045; p < 0.001; 95% CI -0.067 to -0.023) and serum folate (unadjusted β -0.193; p < 0.001; 95% CI -0.295 to -0.091). A positive linear association was observed between the PDR score and tHcy (unadjusted β 0.030; p < 0.001; 95% CI 0.014 to 0.045). Additional adjustment for age, BMI, ethnicity, education, vitamin supplement use and medication use slightly attenuated the effect estimates of the four biomarkers and remained significant.

### Diet and hormonal and metabolic parameters in PCOS patients

In Table 3 the serum characteristics of the PCOS phenotypes are depicted. The PDR score was positively associated with AMH and FAI. A positive linear association was established between the PDR score and AMH (unadjusted β 0.084; p = 0.044; 95% CI 0.002 to 0.165), however after adjustment for age and BMI this association was no longer significant.

**Table 3:** Serum characteristics according to PCOS phenotype.

	HA-PCOS (n = 112)	non HA-PCOS (n = 106)
**Hormonal Parameters:**		
AMH (μg/L)	10.3 (6.7–14.8)	7.6 (4.8–11.2)**
FSH (U/l)	6.1 (5.2–7.2)	5.9 (3.9–7.8)
LH (U/l)	9.8 (6.7–14.6)	6.6 (4.4–11.7)**
Progesterone (nmol/l)	1.2 (0.9–1.9)	1.5 (0.9–10.2)*
17- hydroxyprogesterone (nmol/l)	2.9 (2.2–4.4)	2.7 (1.9–5.8)
Estradiol (pmol/L)	225.5 (181.0–300.5)	234.0 (143.0–363.0)
SHBG (nmol/l)	27.0 (21.4–39.7)	56.4 (45.3–81.6)**
Testosterone (nmol/l)	2.0 (1.6–2.8)	1.2 (0.9–1.6)**
FAI	7.0 (5.5–10.7)	2.2 (1.4–3.0)**
Androstenedione (nmol/l)	11.8 (9.7–17.4)	8.6 (6.4–10.4)**
DHEAS(μmol/L)	5.7 (3.9–7.4)	4.8 (3.3–6.1)**
**Metabolic Parameters:**		
Insulin (pmol/l)	67.0 (40.0–118.0)	34.0 (17.0–57.0)**
Glucose (mmol/l)	4.9 (4.6–5.3)	4.7 (4.5–5.0)**
TG (mmol/L)	1.0 (0.8–1.6)	0.9 (0.7–1.2)*
Total–C (nmol/L)	5.4 (4.6–6.7)	5.2 (4.5–6.7)
HDL-C (nmol/L)	1.4 (1.1–1.8)	1.7 (1.4–2.2)**
LDL-C (nmol/L)	3.8 (3.1–4.6)	3.4 (2.8–4.4)*
Apolipoprotein A1	209.6 (183.3–256.7)	239.4 (198.0–281.8)**
Apolipoprotein B	124.1 (103.6–148.8)	115.2 (84.1–136.1)**

Note: Values are expressed as median (interquartile range)

* = p <0.05

** = p <0.01.

HA = Hyperandrogenic; FSH = Follicle Stimulating Hormone; LH = Luteinizing Hormone; SHBG = Sex Hormone Binding Globulin; FAI = Free Androgen Index; DHEAS = dehydroepiandrosterone sulfate; TG = Triglycerides; Total-C = total Cholesterol; HDL-C = HDL-cholesterol; LDL-C = LDL-cholesterol. Mann Whitney U Tests were performed.

This association was stronger when repeated only in the HA+OD+PCOM and OD+PCOM PCOS patients (unadjusted β 0.109; p = 0.011, 95% CI 0.025 to 0.193) and remained significant after adjustment for age (β 0.100; p = 0.022, 95% CI 0.015 to 0.185) and BMI (β 0.102; p = 0.018, 95% CI 0.018 to 0.186).

The PDR score was also positively associated with FAI (unadjusted β 0.128; p = 0.013; 95% CI 0.028 to 0.229), but lost significance after adjustment for age and BMI.

## Discussion

This explorative study demonstrates that patients with PCOS more often use a self-initiated diet and an inadequate diet than subfertile controls. This is substantiated by the use of a more inadequate diet in patients with the more severe HA phenotype and the positive association between diet inadequacy and the AMH concentration in PCOS patients.

The strength of the study is the large sample size and case-cohort design to reduce selection and information bias. Furthermore, standardized anthropometric measurements were performed by trained counsellors who verified all questionnaire data individually during the preconception visit at the outpatient clinic. Several adjustments were made including BMI to minimize the presence of underreporting and overreporting of dietary intake. The PDR score was previously validated in the same study population and is shown to be a sensitive and simple tool to assess diet inadequacy [25]. Another strength of the study is that biomarkers were measured to validate the PDR score and that all laboratory determinations were measured in the same hospital. The PDR score estimates dietary inadequacy and not unhealthy overconsumption which is a limitation [25]. We are aware that although the PDR score covers the six main food groups, it does not reflect the total nutrient and energy intake. The control group consisted of patients referred for subfertility which reduces the external validity of the study. We were able to use clinical and laboratory data for PCOS diagnosis, which were not available for subfertile controls.

Despite numerous efforts, genetic research does not elucidate the developmental origin of PCOS. We suggest that this can be due to a more significant role of gene-nutrient interactions affecting epigenetic mechanisms of multiple pathways regulating ovarian function during the course of life. Previous research demonstrated that genes are prenatally programmed by androgen exposure in utero, resulting in disturbed ovarian and adrenal steroidogenesis [28], impaired oocyte development [29] and PCOM [30]. The diet is important in epigenetics by supplying substrates and methyl groups that are important for gene methylation, programming and subsequent expression and silencing of the genome. Dietary inadequacies can therefore lead amongst other pathways to derangements in the 1-C pathway, resulting in hypomethylation and altered gene expression [8,31]. As a first hit the nutritional environment in-utero can affect reproductive function in the offspring through impaired gonadal organogenesis before birth [31–33]. In rabbits, Leveille et al showed prenatal and postnatal effects of the diet on ovarian function and morphology by the number of atretic follicles [34]. Not only during early life but also postnatally through puberty and adolescence, diet can affect gene expression as second or third hit influencing disease occurrence and severity. Adolescents suffering from overweight and psychological problems are at risk to develop nutritional inadequacies [17,35]. Therefore these data support our hypothesis that the use of a self-initiated diet and dietary inadequacies can contribute to a clinical heterogeneous spectrum of PCOS phenotype development.

This study showed an association between the use of a more inadequate diet in PCOS patients and disease severity which is further underlined by a lower serum and RBC folate. Homocysteine concentrations were higher as well, although this did not reach statistical significance. The study of Rodrigues et al supports this finding showing a poor diet quality in PCOS patients (56% inadequacy according to the Brazilian healthy eating index) [36]. Studies comparing diet quality in PCOS to controls are contradictory. Comparable dietary compositions in PCOS and controls are reported [37–39], whereas the group of Moran et al observed a higher diet quality in PCOS [40]. A high prevalence of folate deficiency and increased levels of homocysteine is previously observed in PCOS substantiating our results and the association with the involvement of the one carbon pathway [41,42]. An explanation for the different findings in other studies is that in all other studies the assessment of diet inadequacy was performed always after diagnosing PCOS. Moreover, due to the exclusion of dieting, non-stabled weighted or normal weighted PCOS patients in other studies, selection bias could explain the different results. Information bias due to the awareness of PCOS and the intention to treat can also explain the differences with other studies. Finally, in contrast to other studies, we also investigated the association with the severity of the PCOS phenotypes and compared them with subfertile controls.

Our results are in line with studies showing the importance of dietary treatment in PCOS for reproductive and metabolic outcome [16,43,44]. Furthermore, in overweighed PCOS patients it is observed that folate supplement use had beneficial effects on metabolic profiles [45], which indicates the involvement of the 1-C cofactor folate in PCOS.

The positive linear association between the PDR score and AMH concentration, and PCOS severity very much supports the function of AMH as marker of the spectrum [46]. This is supported by Nybacka et al showing a decrease in AMH in PCOS patients following a 4 month calorie restricted, well-balanced diet [16]. Another observation is that PCOS gradually disappears during ageing [47,48]. We suggest that the known improvement of the diet during the course of life also contributes to its disappearance which is supported by the gradual decrease of AMH during ageing [49]. FAI as marker for androgen excess was also positively associated with diet inadequacy, which is in line with the findings of others. [43]. According to the biological gradient criteria of Hill, the associations of AMH and FAI with the PDR score strengthen the relationship between diet inadequacy and PCOS severity. This is substantiated by a stronger linear association between the PDR score and AMH after exclusion of the subpopulation without PCOM (HA+OD) or without OD (HA+PCOM). After adjustment for age and BMI this association remained significant, demonstrating the heterogeneity of the hyperandrogenic phenotype.

To conclude, this explorative study shows for the first time that the use of a self-initiated diet and diet inadequacy are associated with PCOS as well as PCOS phenotype. These findings emphasize the need for prospective research from the reproductive life course onwards with a focus on the role of the inadequacy of the diet in the developmental origins of PCOS. Moreover, in PCOS treatment more attention should be given to the adherence of an adequate diet.

## Supporting Information

S1 Dataset Minimal Dataset. (SAV)

S1 Table Sensitivity analysis. Note: Values are expressed as median (interquartile range) or number (%),* = p <0.05; ** = p <0.01. PDR score = Preconception Dietary Risk score; BMI = Body Mass Index; RBC Folate = Red Blood Cell Folate; tHcy = homocysteine. Normal range biochemical parameters; Folate ≥8 nmol/L, RBC folate ≥500 nmol/L, Cobalamin ≥145 pmol/L, tHcy <15 μmol/L. Chi Square tests and Mann Whitney U Tests were performed.
(DOC)

## Abbreviations

1-C: One-Carbon
AMH: Anti-Müllerian Hormone concentration
BMI: Body Mass Index
FAI: Free Androgen Index
HA: Hyperandrogenism
OD: Ovulatory Dysfunction
PCOM: Polycystic Ovarian Morphology
PCOS: Polycystic Ovary Syndrome
PDR score: Preconception Dietary Risk Score
RBC folate: Red Blood Cell folate
RIA: Radioimmunoassay
SHBG: Sex Hormone-Binding Globulin
tHcy: plasma total homocysteine
