# Supplementary material for: Are Dieting and Dietary Inadequacy a Second Hit in the Association with Polycystic Ovary Syndrome Severity?
Source: PLoS One. 2015 Nov 16;10(11):e0142772. doi: 10.1371/journal.pone.0142772 (PMC4646482; doi:10.1371/journal.pone.0142772)
Supplement: S1 Table — Note: Values are expressed as median (interquartile range) or number (%),* = p <0.05; ** = p <0.01. PDR score = Preconception Dietary Risk score; BMI = Body Mass Index; RBC Folate = Red Blood Cell Folate; tHcy = homocysteine. Normal range biochemical parameters; Folate ≥8 nmol/L, RBC folate ≥500 nmol/L, Cobalamin ≥145 pmol/L, tHcy <15 μmol/L. Chi Square tests and Mann Whitney U Tests were performed. (DOC) [file pone.0142772.s002.doc]

**Supplemental Table I. Sensitivity analysis**

|  | **Study population**  (n=1017) | **Excluded population**  (n=234) |
| --- | --- | --- |
| **Age** (years) | 32.1 (28.4 -35.8) | 33.1 (29.6-37.2)** |
| **Ethnicity** Dutch | 567 (56.0%) | 122 (53.5%) |
| Other | 446 (44.0%) | 106 (46.5%) |
| **Educational level** Low | 150 (15.5%) | 31 (14.2%) |
| Intermediate | 433 (44.8%) | 103 (47.2%) |
| High | 383 (39.6%) | 84 (38.5%) |
| **Lifestyle Parameters:** |  |  |
| Diet (Yes) | 148 (14.6%) | 38 (16.5%) |
| PDR score (mean; sd) | 3.54 (1.14) | 3.47 (1.20) |
| Folic acid supplement use (No) | 360 (35.4%) | 89 (38.0%) |
| Vitamin supplement use (No) | 616 (61.2%) | 129 (55.6%) |
| Medication use (Yes) | 330 (32.6%) | 78 (33.9%) |
| Alcohol (Yes) | 540 (53.1%) | 100 (42.7%)** |
| Smoking (Yes) | 226 (22.5%) | 35 (15.2%)* |
| Physical exercise (No) | 464 (49.7%) | 110 (50.5%) |
| Stress (Yes) | 309 ( 33.7%) | 87 (40.7%) |
| **Measurements:** |  |  |
| BMI (kg/m2) | 24.8 (22.0-28.9) | 24.8 (22.5-28.8) |
| BMI in categories (kg/m2) <20 | 75 (7.4%) | 17 (7.3%) |
| ≥20 <25 | 455 (45.0%) | 109 (46.6%) |
| ≥25 <30 | 280 (27.7%) | 57 (24.4%) |
| ≥30 | 201 (19.9%) | 51 (21.8%) |
| Waist circumference (cm) | 84 (75-94) | 85 (77-97)* |
| Waist hip ratio | 0.81 (0.75-0.87) | 0.82 (0.77-0.90)* |
| **Biochemical Parameters:** |  |  |
| Cobalamin (pmol/L) | 309.0 (236-409) | 313.5 (246-431) |
| RBC Folate (nmol/L) | 1022.0 (817-1303) | 994.5 (815-1268.5) |
| Folate (nmol/L) | 28.5 (18.0 -40.3) | 25.6 (17.8-37.4)* |
| tHcy (µmol/L) | 8.50 (7.1-10.0) | 8.5 (7.1-10.2) |

Note: Values are expressed as median (interquartile range) or number (%),* = p<0.05; ** = p<0.01.

PDR score = Preconception Dietary Risk score; BMI = Body Mass Index; RBC Folate = Red Blood Cell Folate; tHcy = homocysteine. Normal range biochemical parameters; Folate ≥8 nmol/L, RBC folate ≥500 nmol/L, Cobalamin ≥145 pmol/L, tHcy <15 µmol/L. Chi Square tests and Mann Whitney U Tests were performed.
